# Recent Advances and Sustainability Perspectives of Biobased Wood Panel Adhesives: Toward Cleaner and Formaldehyde-Free Wood Products

**DOI:** 10.3390/polym18131672

**Published:** 2026-07-06

**Authors:** Sogand Ghafari Movahed, Iman Rezvani, Ali Dorieh, Saeed Kamrani, Meysam Mehdinia, Mohammadreza Pourpilehkesh, Mohammad Hassan Shahavi, Sara Nabipoor, Petar Antov, Viktor Savov, Viktoria Dudeva, Widya Fatriasari, Lee Seng Hua, Antonio Pizzi

**Affiliations:** 1 School of Chemistry, College of Science, University of Tehran, Tehran 14179-35840, Iran; s.ghafarimovahed@ut.ac.ir; 2 Department of Wood and Paper Science and Technology, Faculty of Natural Resources, University College of Agricultural & Natural Resources, University of Tehran, Karaj 77871-31587, Iran; iman.rezvani@ut.ac.ir (I.R.); mmsd_22@yahoo.com (S.K.); mohammadrezapourpilehkesh@gmail.com (M.P.); 3 Department of Nanotechnology, Faculty of Engineering Modern Technologies, Amol University of Special Modern Technologies (AUSMT), Amol 46156-64616, Iran; ali.dorieh@yahoo.com (A.D.); m.shahavi@ausmt.ac.ir (M.H.S.); 4 Institute of Forest and Rangelands, Agricultural Research Education and Extension Organization (AREEO), Tehran 14968-13111, Iran; meysammehdinia@gmail.com; 5 Science, Tonekabon Branch, Islamic Azad University, Tonekabon 46841-61167, Iran; nabipoor1370@gmail.com; 6 Department of Mechanical Wood Technology, Faculty of Forest Industry, University of Forestry, 1797 Sofia, Bulgaria; v.dudeva@ltu.bg; 7 Research Center for Biomass and Bioproducts, Research Organization for Nanotechnology and Material, National Research and Innovation Agency (BRIN), Serpong 15314, Indonesia; widy003@brin.go.id; 8 Department of Wood Industry, Faculty of Applied Sciences, Universiti Teknologi MARA Pahang Branch Jengka Campus, Bandar Tun Abdul Razak 26400, Pahang, Malaysia; leesenghua@uitm.edu.my; 9 Institute for Infrastructure Engineering and Sustainable Management (IIESM), Universiti Teknologi MARA, Shah Alam 40450, Selangor, Malaysia; 10 LERMAB-ENSTIB, University of Lorraine, 27 rue Philippe Seguin, 88000 Epinal, France

**Keywords:** biobased wood adhesives, wood-based panels, lignin, tannin, starch, soy protein, 5-hydroxymethylfurfural, citric acid, formaldehyde-free adhesives, industrial scalability

## Abstract

Biobased wood adhesives are essential to reducing the dependence of wood-based panels on petrochemical and formaldehyde-emitting resins. This review critically synthesizes recent progress in lignin-, tannin-, starch-, furan/HMF-, organic acid-, and soy protein-based adhesive systems, with emphasis on chemical reactivity, curing mechanisms, water resistance, processability, and industrial relevance. The discussion distinguishes laboratory performance from industrial feasibility by considering specific press time, solids content, viscosity, raw material variability, emissions, cost, life-cycle performance, and compatibility with particleboard, medium-density fibreboard, plywood, and related engineered wood products. Lignin and tannins are highlighted as the most chemically compatible phenolic platforms, starch and soy systems as abundant but moisture-sensitive binders requiring targeted crosslinking, HMF and furan derivatives as promising aldehyde-type formaldehyde-free crosslinkers, and citric acid systems as attractive polyester-forming binders with pressing-temperature limitations. The review concludes that near-term adoption will most likely proceed through hybrid and partially biobased systems, whereas fully biobased adhesives require faster curing, standardized feedstocks, pilot-scale validation, and transparent techno-economic and life-cycle assessment.

## 1. Introduction

Wood adhesives govern the mechanical performance, emissions, processing window, and cost of plywood, particleboard (PB), oriented strand board (OSB), medium-density fibreboard (MDF), and high-density fibreboard (HDF) [1,2,3]. In current industrial practice, formaldehyde-based adhesives such as UF, PF, MF/MUF and their modified variants remain dominant because they combine high reactivity, stable processing and competitive cost [1,2,3,4,5,6,7]. Isocyanate-based and other reference systems are also used where higher moisture resistance or specific performance is required [2,8]. This established industrial baseline makes any transition to biobased binders dependent not only on chemistry but also on press time, resin handling and quality consistency [9,10].

The drive toward cleaner adhesive technologies is motivated first by formaldehyde exposure, toxicity and indoor air quality concerns [5,6,7]. It is also reinforced by regulatory and chemical policy pressure on conventional formaldehyde-emitting systems [8,11]. A second driver is the need to diversify raw material sources and valorise lignocellulosic side streams in a circular bioeconomy [9,10,12]. Recent work on biocrosslinking and dual-network bioadhesive routes illustrates how cleaner systems can be designed at the molecular level [13,14]. Tannin platforms, renewable material supply chains and environmental assessments further support the transition toward lower-emission and more sustainable adhesive systems [15,16,17,18,19]. Biobased adhesives are therefore not only an environmental topic but also a manufacturing challenge because any new binder must fit the speed, moisture tolerance, and variability of industrial wood panel lines.

The present review differs from broad descriptive reviews by focusing on the gap between laboratory demonstrations and industrial implementation, addressing the need for critical evaluation emphasized in recent bioadhesive reviews [20,21]. Environmental and life-cycle studies show that a formulation cannot be judged only by laboratory strength data [22,23,24]. Techno-economic analysis is also needed to assess whether reported adhesive routes can compete with established systems at scale [25]. The review therefore evaluates whether reported formulations provide adequate bonding and durability under realistic process constraints, particularly specific press time, curing temperature, viscosity, solids content, storage stability, and feedstock consistency.

## 2. Scope and Critical Evaluation Framework

The review covers renewable or partly renewable adhesive systems based on lignin, tannins, starch, furan platform molecules such as 5-hydroxymethylfurfural (HMF), naturally occurring organic acids, and soy proteins. Petroleum-derived systems are discussed only when they serve as reference resins or as co-reactants in hybrid formulations.

A recurring weakness in the literature is the direct comparison of laboratory-made boards with quality standards without sufficient attention to processing. Meeting a mechanical requirement after very long pressing, unusually high temperature, elevated resin loading, or selected raw material does not necessarily indicate industrial readiness. For this reason, this review gives higher weight to studies that report specific press time, adhesive solids content, mat moisture, resin loading, panel density, board thickness, and durability testing.

## 3. Major Adhesive Systems

The following sections summarize the principal biobased platforms from a structure–processing–property perspective, identifying promising chemistries, unresolved limitations, and realistic near-term applications.

### 3.1. Lignin-Based Adhesives

Lignin is the most abundant aromatic biopolymer and is attractive because its phenolic structure resembles part of PF chemistry [26,27,28,29,30,31,32,33,34,35]. Technical lignins from kraft, sulfite, soda, organosolv and biorefinery streams differ strongly in molecular weight, sulfur content, solubility, hydroxyl functionality and condensation degree [31,32,33]. These differences explain why lignin performance varies more than that of petrochemical phenol and why valorisation routes require careful matching of lignin type, modification strategy and final adhesive use [27,29,30]. Market, sustainability and application-oriented studies further show that lignin feasibility depends on cost, processing route and supply consistency, not only on theoretical aromatic availability [34,36,37,38,39].

Unmodified technical lignin generally has low reactivity and slow curing, which remain among the main barriers to direct adhesive use [29,37,39]. Useful adhesive routes therefore include methylolation and phenolation to increase reactive sites [40,41,42], demethylation or oxidation to increase phenolic hydroxyl availability [43], and depolymerization or fractionation to improve mobility and processability [27,44,45]. Grafting and epoxidation represent additional strategies for introducing reactive groups suitable for crosslinked adhesive networks [46]. In PF-type systems, lignin can partly substitute for phenol, but the acceptable substitution level depends on the lignin source and the required pressing speed.

Recent studies have expanded lignin chemistry toward methylated, methylolated, glyoxal- and HMF-based systems [47,48,49,50]. Epoxy-modified lignin and lignin-based polyurethane/isocyanate routes represent another group of approaches aimed at higher crosslink density and water resistance [51,52,53]. Enzymatic activation, lignin conversion strategies, nanocellulose- or lignin-containing hybrid adhesives, and vitrimer-type networks have also been reported [54,55,56,57]. Additional studies emphasize lignin supply, glyoxal networks, nanolignin modification and chitosan-containing systems for panel applications [58,59,60,61]. These routes can improve water resistance and bonding strength, yet many still rely on expensive crosslinkers, long curing, or chemicals that weaken the claim of full biobased character.

For panels, lignin is most credible in three near-term uses. First, kraft lignin binders and industrial lignin-based systems can act as partial phenol or urea substitutes [62,63]. Second, lignin can serve as a biobased additive or emission-reducing component in particleboard, hardboard, MDF and related composites [64,65,66,67,68]. Third, lignin–furan/lignosulfonate additives and lignin–urea–formaldehyde systems offer one pathway toward more reactive formulations [69,70,71,72]. Lignin–poly(vinyl alcohol)/hexamine and lignin-based non-isocyanate polyurethane networks represent another group of adhesive systems [73,74]. The critical industrial question is whether these systems can reach the required properties at competitive specific press times and with reproducible technical lignin streams.

**Figure 1:**
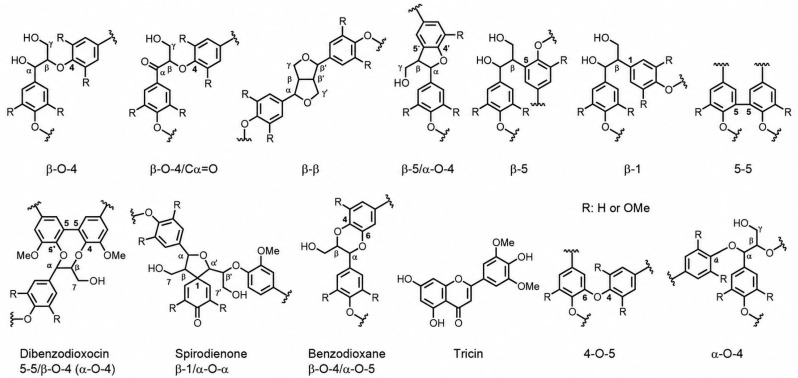
Main interunit linkages in a representative lignin fragment, illustrating the heterogeneous aromatic network relevant to lignin adhesive reactivity. Reproduced with permission from Lignin Valorization: Emerging Approaches; published by the Royal Society of Chemistry, 2018 [35].

The heterogeneous, highly branched molecular structure of lignin is illustrated in Figure 1, showing representative interunit linkages. This structural complexity is directly relevant to lignin-based adhesive formulations, as the type and distribution of linkages, functional groups, and aromatic units strongly influence lignin reactivity, modification efficiency, curing behaviour, and bonding performance.

**Figure 2:**
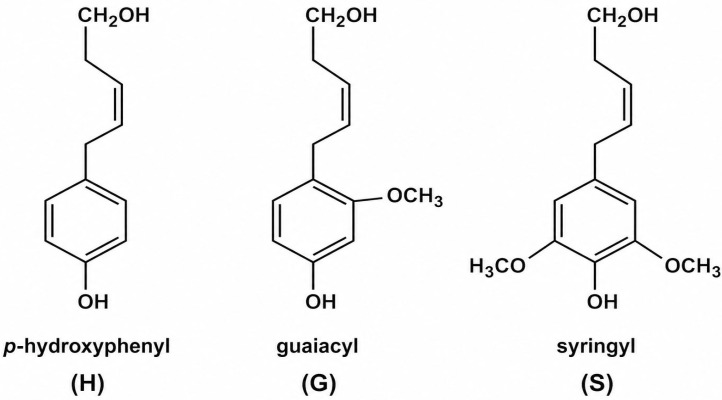
Principal phenylpropanoid lignin units: p-hydroxyphenyl (H), guaiacyl (G), and syringyl (S). Drawn by the authors based on the generally accepted structural representation of lignin monomeric units [26,36].

The three principal phenylpropanoid units of lignin—p-hydroxyphenyl, guaiacyl, and syringyl—are presented in Figure 2. Their relative abundance varies with biomass source and pulping process, affecting the availability of reactive phenolic sites and, consequently, the suitability of different technical lignins for adhesive formulations.

**Figure 3:**
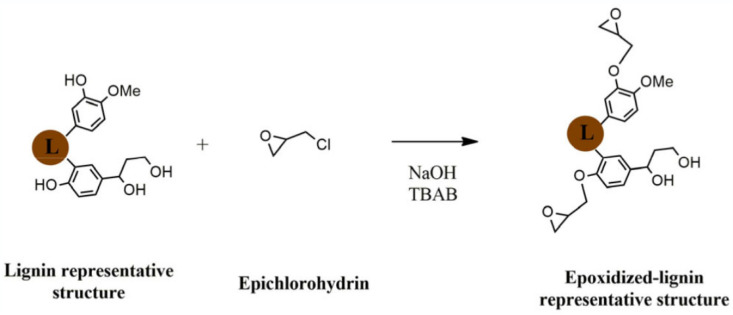
Epoxidation of kraft lignin through reaction of phenolic groups with epichlorohydrin under alkaline conditions [52].

Among the chemical modification strategies used to enhance lignin reactivity, epoxidation is particularly important because it converts part of the phenolic hydroxyl functionality of lignin into epoxy-bearing structures with higher crosslinking potential. This modification can improve the compatibility of lignin with epoxy-type adhesive systems and increase the number of reactive sites available for network formation during curing. As shown in Figure 3, kraft lignin can be epoxidized through the reaction of its phenolic groups with epichlorohydrin under alkaline conditions. The resulting epoxidized lignin structure represents a more reactive adhesive precursor, which may contribute to improved bonding strength, enhanced water resistance, and broader applicability of lignin in formaldehyde-free wood adhesive systems. However, the practical relevance of this route still depends on reaction efficiency, economic costs, toxicity concerns associated with epichlorohydrin, and the ability of the modified lignin system to cure under industrially acceptable pressing conditions.

### 3.2. Tannin-Based Adhesives

Tannins are among the most mature renewable phenolic adhesive raw materials, with extensive research and review literature already available [75,76]. Condensed tannins from mimosa, quebracho, pine and other bark sources possess highly reactive flavonoid units that can undergo electrophilic substitution and self-condensation [77,78,79]. Their high reactivity explains why tannin adhesives can cure with formaldehyde, hexamine, glyoxal, furfuryl alcohol or other aldehyde-type and furanic crosslinkers [77,80,81].

The chemical strength of tannins is also their processing challenge. High viscosity, fast pot life reduction, regional variability, dark colour and limited extract availability can restrict industrial use [82,83,84,85,86]. Studies on tannin resin chemistry, rearrangement/self-condensation and chestnut shell tannins provide further evidence for the importance of source-specific chemistry [87,88,89,90,91,92,93]. Nevertheless, tannin-based adhesives have demonstrated realistic potential for particleboard and MDF when the extract source and hardener system are properly selected [82,83,85,90,91]. Soy–tannin, lignin-derived aldehyde, tannin–furfuryl alcohol and polyethyleneimine-improved formulations illustrate how crosslinker choice can improve performance [88,94,95,96,97]. Non-isocyanate polyurethane and patent-level developments indicate that tannin chemistry is also expanding beyond classical phenolic-type adhesives [77,98,99].

**Figure 4:**
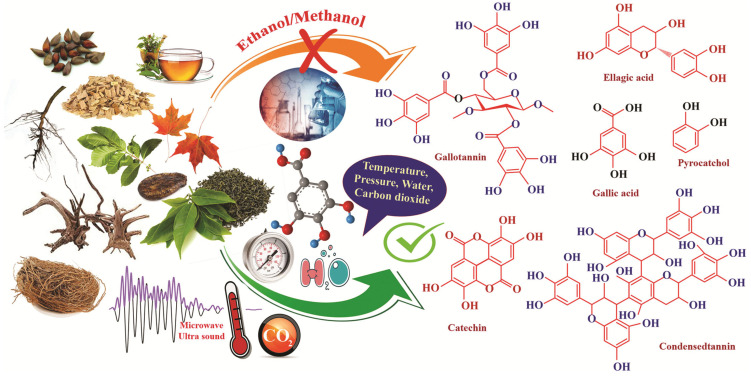
Extraction routes and major phenolic constituents obtained from tannin-rich renewable resources [15].

The diversity of tannin-rich biomass resources and extraction approaches is summarized in Figure 4, highlighting the range of phenolic constituents that can be obtained from renewable natural feedstocks. This compositional diversity is important because tannin source, extraction conditions, and the presence of non-tannin components strongly affect adhesive reactivity, viscosity, curing behaviour, and final bonding performance.

**Figure 5:**
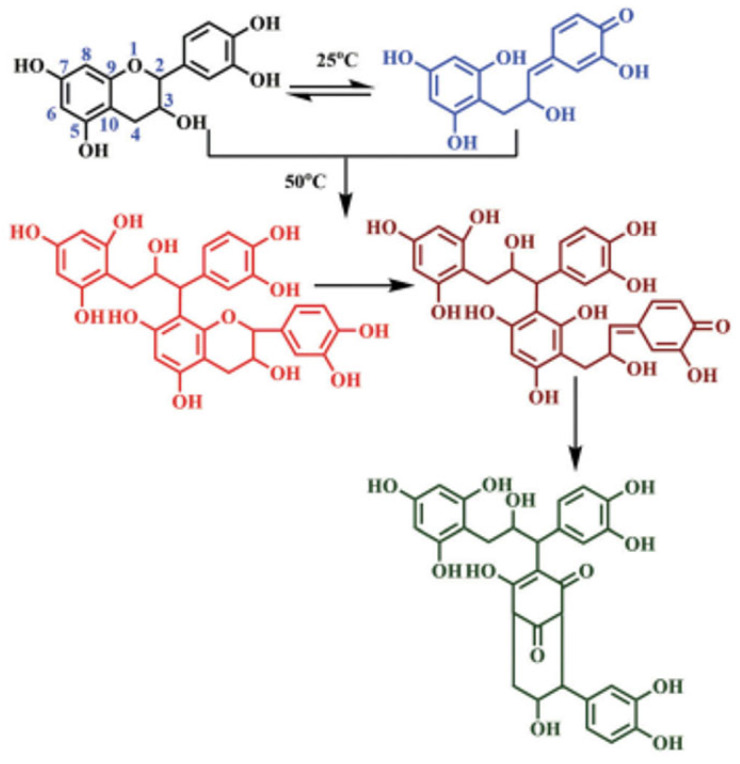
Simplified pathway for tannin self-condensation and formation of polymeric structures [15,92].

Beyond their reaction with external crosslinkers, condensed tannins can also participate in self-condensation reactions, contributing to the formation of polymeric networks during curing. A simplified representation of this pathway is presented in Figure 5, illustrating how tannin structures can generate higher-molecular-weight adhesive networks relevant to wood bonding.

**Figure 6:**
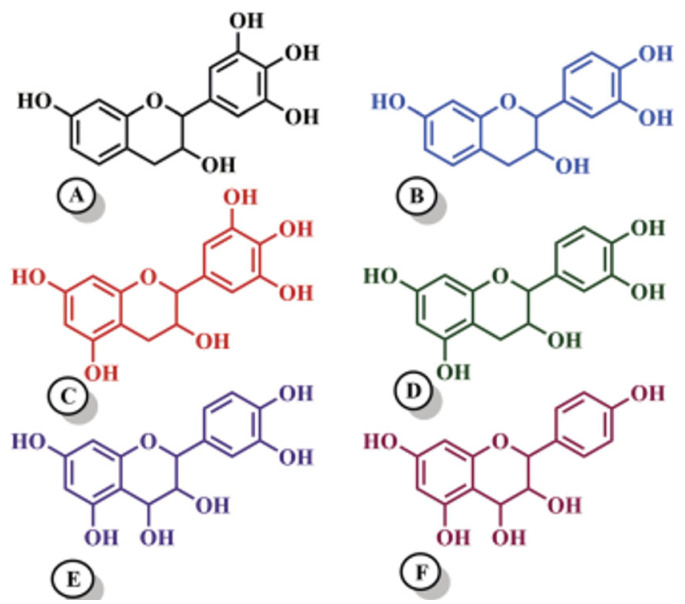
Representative condensed tannin units and non-tannin components relevant to reactivity and network formation: (**A**) robinetinidin-type unit, (**B**) fisetinidin-type unit, (**C**) delphinidin-type unit, (**D**) catechin-type unit, and (**E**,**F**) non-tannin compounds present in tannin extracts [15].

The adhesive performance of condensed tannins is strongly governed by their molecular structure and by the presence of accompanying non-tannin components in the extract. As illustrated in Figure 6, condensed tannins are composed of flavonoid units with multiple phenolic hydroxyl groups and reactive aromatic positions, which enable rapid condensation reactions and network formation during curing. At the same time, non-tannin constituents such as gums, sugars, and other extractives may influence viscosity, solubility, pot life, and final bond durability. Therefore, understanding the structural composition of tannin extracts is crucial for optimizing tannin-based adhesive formulations, particularly when selecting suitable crosslinkers and adjusting processing parameters for wood-based panel production.

**Figure 7:**
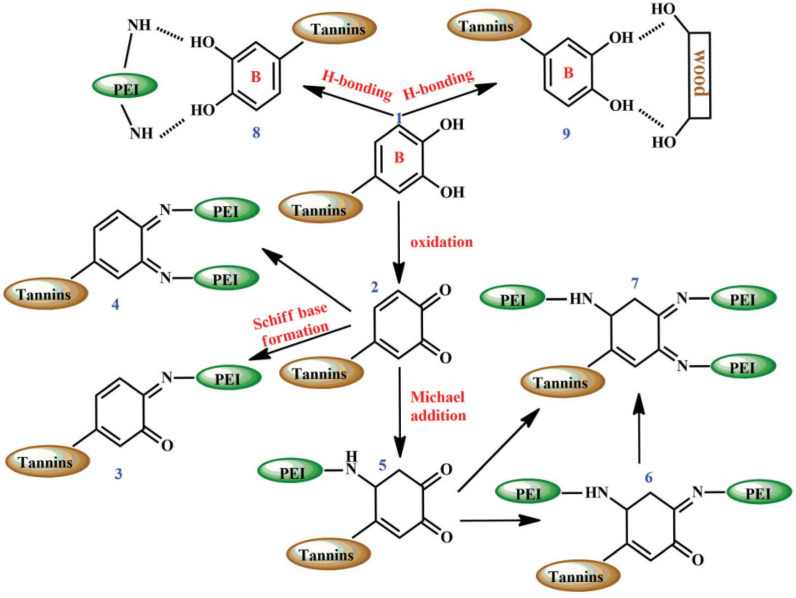
Potential hydrogen bonding interactions between condensed tannin, polyethyleneimine (PEI), and wood hydroxyl groups. Adapted from Dhawale et al. [15].

In addition to covalent condensation reactions, tannin-based adhesive systems can also benefit from extensive secondary interactions with both modifiers and wood substrates. Figure 7 illustrates the potential hydrogen bonding interactions between condensed tannin, polyethyleneimine (PEI), and wood hydroxyl groups. These interactions are important because the numerous phenolic hydroxyl groups of tannins can form hydrogen bonds with the hydroxyl-rich surface of wood, while PEI can provide additional amino functionality that improves interfacial adhesion and network cohesion. Such non-covalent interactions may contribute to improved wetting, stronger adhesive–wood contact, and enhanced bond strength, particularly when combined with chemical crosslinking reactions during curing. Therefore, the use of PEI or similar multifunctional modifiers represents a promising route to improve the performance of tannin-based adhesives while maintaining a largely biobased adhesive framework.

**Figure 8:**
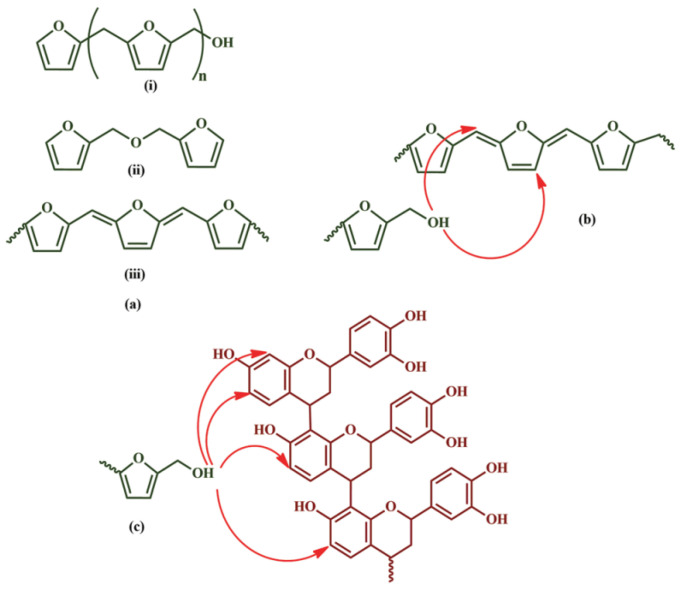
Furfuryl alcohol self-condensation pathways and possible crosslinking sites with tannin flavonoid units: (**a**) representative furfuryl alcohol self-condensation products, including (**i**) a poly(furfuryl alcohol)-type chain, (**ii**) an ether-linked furan dimer, and (**iii**) a conjugated furan oligomer; (**b**) reactive sites involved in further furan network formation; and (**c**) possible reaction/crosslinking sites between furfuryl alcohol-derived fragments and tannin flavonoid units. Adapted from Abdullah and Pizzi [96] and Dhawale et al. [15].

Furfuryl alcohol is an important furan-derived modifier for tannin-based adhesive systems because it can undergo acid-catalysed self-condensation while also participating in reactions with reactive sites on tannin flavonoid units. As shown in Figure 8, the self-condensation of furfuryl alcohol can generate polymeric furan networks, while its interaction with tannin structures may contribute to additional crosslinking and improved adhesive cohesion. This combined tannin–furan chemistry is particularly relevant for developing formaldehyde-free or low-formaldehyde wood adhesives with enhanced water resistance and network stability.

### 3.3. Starch-Based Adhesives

Starch is abundant, inexpensive and biodegradable, but native starch is hydrophilic and forms adhesive films with limited wet strength [100,101,102,103]. Its usefulness in wood panels therefore depends on chemical modification, including Schiff base formation, oxidation and hydrophobization strategies [104,105,106,107]. Film reinforcement and rheology control can also be achieved through lignin addition, cellulose reinforcement, crosslinking or borate chemistry [108,109,110,111]. In panel applications, performance is further influenced by surfactants, silane coupling agents and processing conditions [112,113,114,115,116].

**Figure 9:**
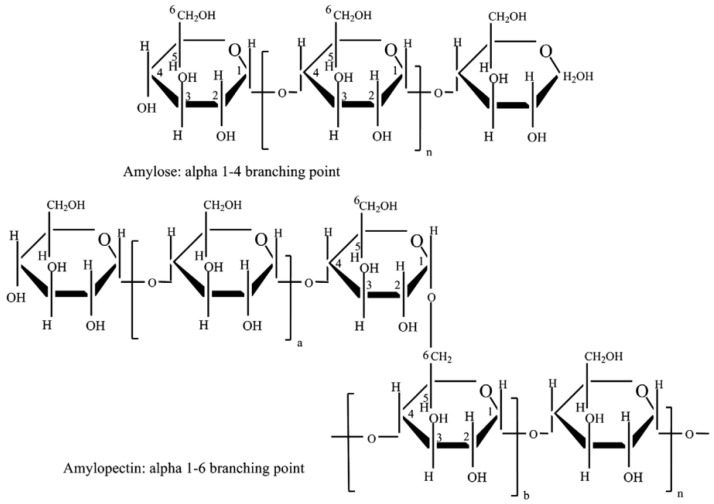
Amylose and amylopectin as the two main structural components of starch. Reproduced with permission from Din et al., Starch; published by John Wiley and Sons, 2020 [100].

The adhesive behaviour of starch is closely related to its molecular composition, particularly the relative proportions and structural arrangement of amylose and amylopectin. As shown in Figure 9, amylose is mainly a linear polysaccharide, whereas amylopectin has a highly branched architecture. These structural differences influence gelatinization, viscosity, film formation, water sensitivity, and the availability of hydroxyl groups for chemical modification or crosslinking. Therefore, understanding the basic structure of starch is essential for designing starch-based wood adhesives with improved bonding performance, reduced hydrophilicity, and enhanced resistance to moisture. Modification strategies such as oxidation, esterification, grafting, or multifunctional crosslinking are largely aimed at overcoming the limitations associated with the native hydrophilic starch structure.

From an industrial viewpoint, starch adhesives are most realistic for interior or low-moisture applications, for hybrid formaldehyde-free binders, and for systems where cost and low toxicity outweigh the demand for structural wet durability. Reported improvements in water resistance should be interpreted together with adhesive loading, panel density, board thickness, and press time [112,113,114,115,116].

**Figure 10:**
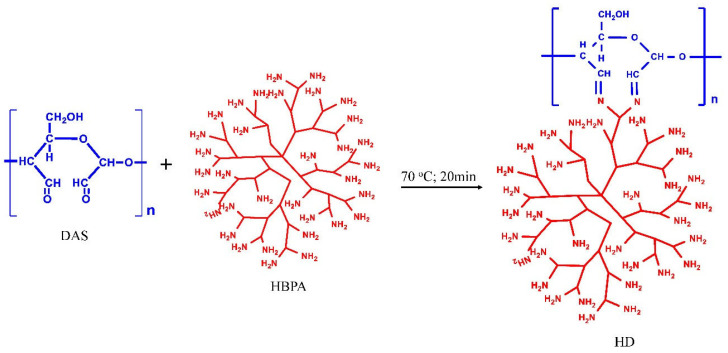
Grafting mechanism of hyper-branched aminated starch (HD) from dialdehyde starch (DAS) and hyper-branched polyamide (HBPA). Adapted from Zhang et al. under the terms of the Creative Commons CC BY license [117].

Chemical modification and hybridization are commonly used to overcome the intrinsic moisture sensitivity and limited wet strength of native starch adhesives [107,117]. In this context, Figure 10 illustrates the grafting mechanism of hyper-branched aminated starch (HD), obtained through the reaction of dialdehyde starch (DAS) with hyper-branched polyamide (HBPA). The aldehyde groups introduced into the starch backbone can react with amino groups in HBPA through Schiff base chemistry, resulting in a multifunctional, amino-rich, hyper-branched starch derivative. Such modification increases the number of available reactive sites and can contribute to higher crosslinking density, improved cohesion, and better water resistance in starch- or starch-containing adhesive systems.

### 3.4. Furan and 5-Hydroxymethylfurfural-Based Adhesives

HMF is a biomass-derived platform molecule obtained through carbohydrate dehydration [118,119]. Its aldehyde, hydroxymethyl and furan functionalities make it attractive as a formaldehyde-free crosslinker for lignin, tannin, carbohydrate and furan–acetone systems [120,121]. Scale-up studies and HMF-based thermoset composite work further support its potential while also highlighting the need for stable precursor quality and controlled curing behaviour [122,123]. Related humin-based thermoset adhesive systems, including non-isocyanate polyurethane routes, further illustrate the broader potential of carbohydrate-derived platform products in formaldehyde-free wood adhesive development [124].

**Figure 11:**
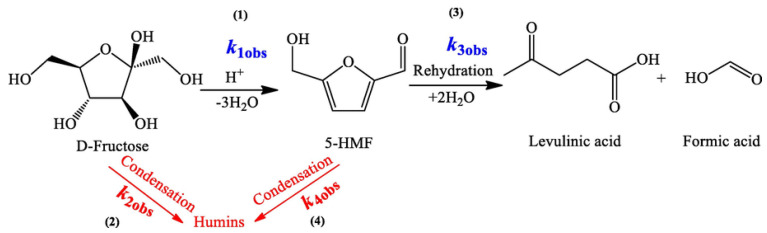
Dehydration of fructose to 5-hydroxymethylfurfural (5-HMF) and side formation of formic and levulinic acids. Adapted from the original publication [125] and from Aranha and Gogate [118] and Teong et al. [119].

The chemical relevance of HMF as a biobased adhesive intermediate is closely connected to its formation from carbohydrates and to the reactive functional groups retained in its molecular structure. As shown in Figure 11, 5-hydroxymethylfurfural (5-HMF) can be produced through the dehydration of fructose, with formic and levulinic acids formed as possible side products. The presence of both aldehyde and hydroxymethyl groups, together with the furan ring, gives HMF aldehyde-type reactivity without relying on formaldehyde. This makes it a promising crosslinker for lignin-, tannin-, carbohydrate-, and furan-based adhesive systems.

The value of HMF is not simply its renewable origin but its ability to provide aldehyde-type reactivity without using formaldehyde. The main unresolved issues are production cost, purification, colour development, side reactions, storage stability, and the need to demonstrate curing at industrially relevant specific press times.

### 3.5. Naturally Occurring Organic Acid-Based Adhesives

Citric acid and related organic acid systems bond wood mainly through esterification with hydroxyl groups in cellulose, hemicelluloses and lignin [126,127,128]. They are attractive because they are formaldehyde-free and can be combined with sugars or sucrose for particleboard and related wood composite formulations [129,130,131]. Citric acid systems have also been applied with glycerol, starch or oil palm-based components in plywood, MDF and other wood composites [132,133,134]. Foundational and review studies confirm the role of citric acid as a green binder or modifying agent but also show that pressing temperature and moisture management remain critical [127,135,136,137].

**Figure 12:**
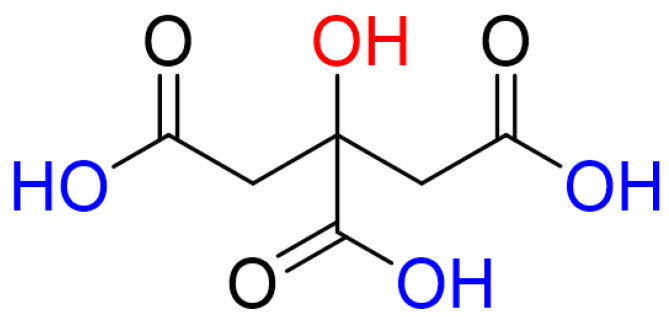
Chemical structure of citric acid, illustrating the multifunctional carboxyl and hydroxyl groups relevant to esterification-based bonding in wood panels. Adapted from [137] under the terms of the Creative Commons CC BY license.

The adhesive function of citric acid is directly related to its molecular structure and the presence of multiple carboxyl groups. As shown in Figure 12, citric acid contains three carboxylic acid groups and one hydroxyl group, which enable esterification reactions with the hydroxyl groups present in wood polymers such as cellulose, hemicelluloses, and lignin. This multifunctional structure allows citric acid to act not only as a biobased crosslinking agent but also as a formaldehyde-free binder capable of forming polyester-type networks during hot pressing. These reactions are particularly important for improving the dimensional stability and water resistance of wood-based panels.

The main limitation is processing. Effective esterification often requires sufficiently high temperatures, moisture removal, and pressing periods that may be longer than industrial PB or MDF targets. Excess acidity can also increase brittleness, corrosion risk, or degradation of carbohydrates. Therefore, promising future directions include buffered or self-neutralizing formulations and hybrid organic acid systems that lower curing severity.

**Figure 13:**
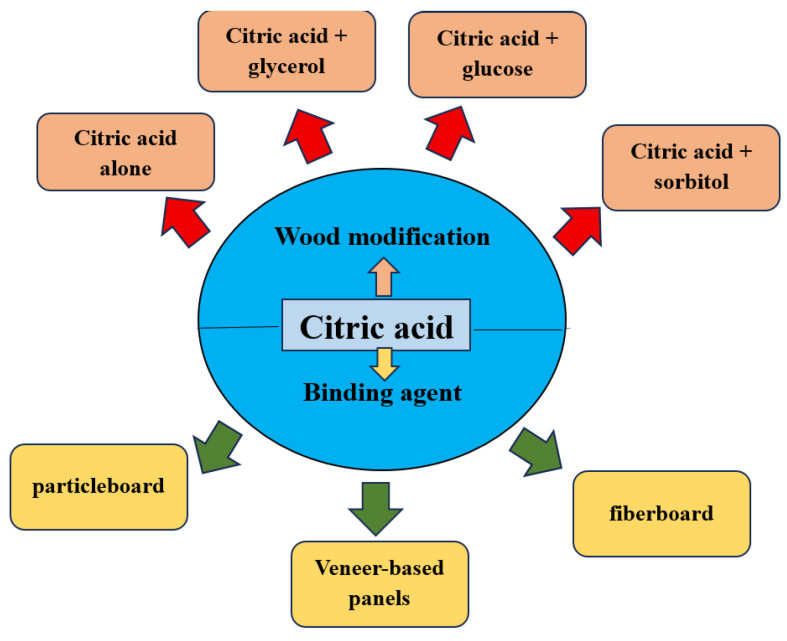
Citric acid as a wood-modifying and binding agent for wood-based panels [137].

Figure 13 highlights both the potential and the processing sensitivity of citric acid-based adhesive systems for wood-based panel production. It also underlines the need to balance curing efficiency, bond durability, dimensional stability, and energy consumption when optimizing these systems for industrial-scale wood panel manufacturing.

### 3.6. Soy Protein-Based Adhesives

Soy adhesives are based mainly on defatted soy flour or soy protein isolate, and they have been widely reviewed for wood bonding and particleboard applications [138,139,140,141]. Their amino, carboxyl, hydroxyl and sulfhydryl groups provide multiple reaction pathways, but the same polar functionality causes high water sensitivity unless the protein is denatured, unfolded or crosslinked [142,143,144].

**Figure 14:**
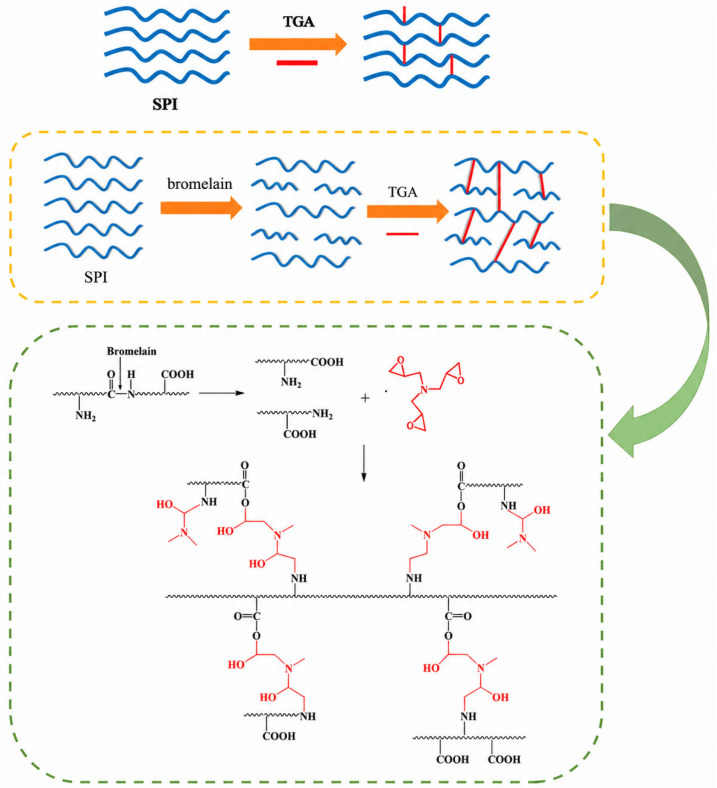
Enzymatic hydrolysis mechanism of soybean protein adhesives [143].

The performance of soy protein-based adhesives depends strongly on the molecular structure and accessibility of reactive protein groups. Enzymatic hydrolysis is one strategy used to modify soy proteins by partially breaking peptide chains, thereby exposing additional amino, carboxyl, hydroxyl, and other functional groups that can participate in bonding and crosslinking reactions. As illustrated in Figure 14, controlled enzymatic hydrolysis can improve protein dispersion, increase molecular mobility, and facilitate interactions with wood surfaces or added crosslinkers. This modification may contribute to improved adhesive penetration, stronger interfacial bonding, and enhanced curing efficiency.

Improved soy adhesives commonly use dual crosslinking, cardanol-based epoxy compounds, glucose/citric acid networks, lignin–protein hybridization or other biobased modifiers [145,146,147,148,149]. Gum arabic, sorghum lignin, condensed tannins and classical soy crosslinking routes further demonstrate the versatility of soy-based adhesive chemistry [150,151,152,153]. These strategies increase crosslink density, reduce hydrophilicity and improve wet bonding, but they may also increase cost, viscosity and formulation complexity. In wood panel studies, performance has also been assessed for MDF, particleboard and UF/soy hybrid systems [154,155,156,157].

Soy systems are most credible for formaldehyde-free plywood, MDF, and selected PB applications where interior service conditions, product value, or emission requirements justify slower curing or higher adhesive loading. Pilot-scale validation remains essential because many positive laboratory results were obtained under processing conditions that are not directly transferable to continuous lines.

**Figure 15:**
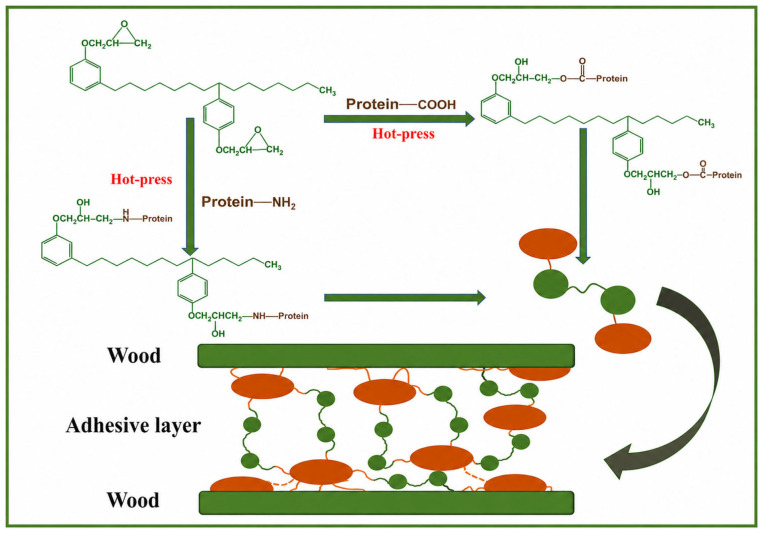
Proposed curing reactions in soybean protein-based adhesives. Adapted from Zhu et al. [146].

The curing performance of soy protein-based adhesives is governed by the availability of reactive functional groups and their ability to form a stable crosslinked network during hot pressing. As shown in Figure 15, soybean protein can participate in several curing reactions involving amino, carboxyl, hydroxyl, and other polar groups, either through self-interactions or reactions with added crosslinkers and modifiers. These reactions are essential for improving cohesive strength, reducing water sensitivity, and enhancing the durability of the adhesive bond. The proposed curing pathways also demonstrate why protein unfolding, denaturation, and chemical modification are often necessary to expose hidden reactive sites and promote more effective network formation.

**Figure 16:**
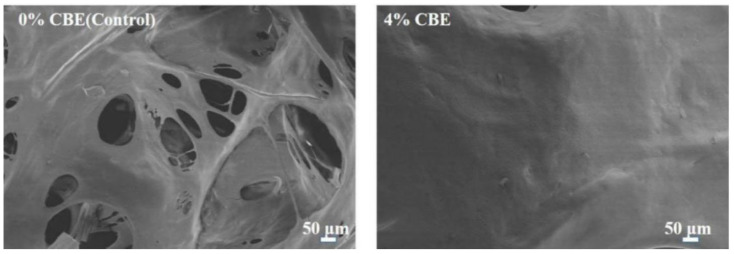
SEM fracture surfaces of cured soybean protein-based adhesives. Adapted from Zhu et al. [146].

The morphology of cured soy protein-based adhesive networks provides important evidence for understanding how denaturation, chemical modification, and crosslinking affect adhesive structure and bonding performance [146,158]. As shown in Figure 16, SEM observations of fracture surfaces can reveal differences in compactness, continuity, pore formation, homogeneity, and the presence of cracks or voids within the cured adhesive. The unmodified soybean protein adhesive exhibits a more porous and discontinuous fracture surface, whereas the adhesive modified with cardanol-based epoxy (CBE) shows a denser and more continuous morphology. Such morphological densification indicates improved compatibility among adhesive components, more effective network formation, and stronger cohesive strength. It can also reduce pathways for water penetration and contribute to improved wet bonding performance. In contrast, loose, porous, cracked, or discontinuous fracture surfaces may indicate incomplete curing, insufficient crosslinking, or weaker interactions between adhesive components. Similar structure–property relationships have also been reported for other chemically modified soy protein-based adhesive systems [146].

## 4. Industrial Processing and Standard-Based Interpretation

The strongest criticism of many biobased adhesive studies is not that the reported panels perform poorly, but that they are evaluated without a realistic processing context. A board may satisfy a standard after long pressing, high resin content, selected particles, or favourable density, yet still be commercially unattractive [1,2,9,10]. Specific press time is particularly important because industrial productivity is linked to seconds per millimetre rather than only to total press duration, and this parameter is rarely comparable across laboratory studies [2,9,25].

Therefore, the comparison with standards should be framed as a first screening rather than a final proof of feasibility. For interior PB and MDF, internal bonds, bending properties, thickness swelling, water absorption, and formaldehyde emission should be reported together with density profile, moisture, resin content, wax addition, and pressing schedule [1,3,8,112,113,114,115,116]. For plywood and structural products, wet shear strength, boiling water resistance, delamination, and durability are essential because these properties determine whether an adhesive can move beyond dry interior applications [2,8,138,139,140,141].

A realistic commercialization path is staged. First, renewable components can be used as extenders, formaldehyde scavengers, or partial substitutes in existing systems, particularly when they improve emissions without strongly affecting curing speed or resin handling [8,9,12,20]. Second, hybrid systems can target specific performance gaps, such as water resistance, curing rate, or compatibility with existing press schedules [13,14,73,88,94,95,96,97]. Third, fully biobased systems should be developed only when they can meet process and durability requirements without excessive cost, energy demand, or formulation complexity [22,23,24,25].

A systematic comparison across adhesive systems shows that lignin and tannin systems are chemically closest to conventional phenolic networks and are therefore the most suitable for partial substitution or hybrid phenolic-type formulations, although lignin variability and tannin viscosity remain important barriers [29,31,32,33,34,35,36,37,38,39,75,76,77,78,79,80,81,82,83,84,85,86]. Starch- and soy protein-based adhesives are more abundant, low-toxicity, and attractive for interior applications, but both require crosslinking or hydrophobization to overcome intrinsic moisture sensitivity [100,101,102,103,104,105,106,107,108,109,110,111,112,113,114,115,116,138,139,140,141,142,143,144,145,146,147,148,149]. HMF/furan and citric acid systems provide promising formaldehyde-free reaction routes, but their industrial relevance depends on precursor cost, curing severity, acidity, and the ability to reach adequate bond performance under realistic pressing conditions [118,119,120,121,122,123,126,127,128,129,130,131,132,133,134,135,136,137]. Thus, the most realistic near-term route is not a direct replacement of UF, PF, MUF, or pMDI, but rather platform-specific hybridization and partial substitution.

## 5. Sustainability, Life-Cycle Performance, and Techno-Economic Aspects

Biobased origin alone does not guarantee environmental superiority. A binder may reduce formaldehyde emissions but increase energy use, chemical input, cost, adhesive loading, or press time [19,22,23,24,25]. Life-cycle assessment and techno-economic analysis should therefore accompany the development of mature formulations, especially when extraction, purification, chemical functionalization, or thermal curing is intensive [22,23,24,25].

The most favourable sustainability profile is expected when the adhesive uses low-value side streams, requires limited purification, cures at moderate temperatures, enables lower emissions, and does not compromise panel durability [9,12,19,22,23,24,25]. Conversely, very high adhesive loading, long hot pressing, expensive crosslinkers, or intensive chemical modification may offset some advantages of renewable feedstocks, particularly for starch-, soy-, lignin-, or HMF-derived systems that require additional activation or crosslinking steps [25,29,37,100,101,102,103,104,105,106,107,108,109,110,111,112,113,114,115,116,138,139,140,141,142,143,144,145,146,147,148,149].

From a comparative sustainability perspective, lignin and tannin systems benefit from lignocellulosic side-stream availability and phenolic reactivity, but their environmental and economic performance depends on extraction, purification, consistency, and modification intensity [9,19,22,23,24,25,31,32,33,34,35,36,37,38,39,75,76,77,78,79,80,81,82,83,84,85,86]. Starch and soy systems benefit from abundance, low toxicity, and biodegradability, but their water sensitivity may require additional chemical inputs that should be included in sustainability assessments [100,101,102,103,104,105,106,107,108,109,110,111,112,113,114,115,116,138,139,140,141,142,143,144,145,146,147,148,149]. HMF/furan and citric acid systems are attractive because they can provide formaldehyde-free curing routes, yet their overall benefit depends on precursor production, curing temperature, side reactions, and process energy demand [118,119,120,121,122,123,126,127,128,129,130,131,132,133,134,135,136,137]. Future studies should therefore report both absolute performance and process-normalized indicators, such as property per adhesive loading, emission reduction per renewable fraction, performance achieved at industrially relevant specific press time, and life-cycle or cost indicators per functional panel unit. Such metrics would allow the field to move from optimistic laboratory demonstrations to comparable technology-readiness assessments.

## 6. Conclusions and Outlook

This review demonstrates that biobased wood adhesives have progressed substantially from laboratory concepts toward more application-oriented systems; however, their technological readiness remains strongly platform-dependent. The systematic comparison presented in this review indicates that each adhesive system has a different readiness profile: lignin and tannins are closest to conventional phenolic chemistry, starch and soy proteins are attractive for low-toxicity and interior applications but require moisture resistance improvement, and HMF/furan and citric acid systems offer promising formaldehyde-free curing routes that still require process optimization. Tannins and lignin, due to their phenolic character, are chemically closest to conventional phenolic networks and currently represent the most mature options for hybrid or partially biobased wood panel adhesives. Starch and soy proteins are abundant, low-toxicity, and economically attractive renewable feedstocks, but their intrinsic hydrophilicity and limited wet durability require targeted crosslinking, modification, or hybridization strategies. HMF/furan derivatives and citric acid-based systems offer promising formaldehyde-free curing routes, yet challenges related to production cost, side reactions, curing severity, acidity, storage stability, and process control still limit their broader industrial implementation.

The main research priority is no longer only to demonstrate that a renewable material can bond wood but to develop adhesive systems that combine strong bonding performance with industrially realistic processing. Future formulations must cure rapidly, tolerate variability in both biomass-derived feedstocks and wood raw materials, provide durable wet performance, reduce formaldehyde and VOC emissions, and remain economically competitive with conventional UF, PF, MUF, and pMDI systems. Pilot-scale validation, long-term durability assessment, and integrated life-cycle and techno-economic analyses should become standard requirements for evaluating mature biobased adhesive technologies.

A realistic near-term transition will most likely rely on hybrid systems, partial substitution strategies, and renewable additives used as extenders, co-reactants, or emission-scavenging components, rather than the immediate full replacement of conventional UF, PF, MUF, or pMDI resins. Fully biobased adhesives remain an important long-term objective, but their industrial adoption will depend on combining renewable chemistry with strong curing behaviour, stable raw material supply, reproducible adhesive quality, compatibility with existing panel production lines, and clear sustainability evidence. From a circular bioeconomy perspective, the greatest value of these adhesive systems lies in their ability to valorise lignocellulosic side streams, reduce dependence on fossil resources, lower harmful emissions, and support the production of cleaner and more recyclable wood-based panels. Overall, the successful commercialization of biobased wood adhesives will require a balanced integration of chemical performance, process efficiency, economic feasibility, and verified environmental benefit within a circular and resource-efficient materials economy.

## Data Availability

The original contributions presented in this study are included in this article. Further inquiries can be directed to the corresponding authors.
